# Bisphosphonate Treatment Beyond 5 Years and Hip Fracture Risk in Older Women

**DOI:** 10.1001/jamanetworkopen.2020.25190

**Published:** 2020-12-07

**Authors:** Monika A. Izano, Joan C. Lo, Annette L. Adams, Bruce Ettinger, Susan M. Ott, Malini Chandra, Rita L. Hui, Fang Niu, Bonnie H. Li, Romain S. Neugebauer

**Affiliations:** 1 Division of Research, Kaiser Permanente Northern California, Oakland; 2 Department of Research and Evaluation, Kaiser Permanente Southern California, Pasadena; 3 Department of Medicine, University of Washington School of Medicine, Seattle; 4 Pharmacy Outcomes Research Group, Kaiser Permanente California, Oakland

## Abstract

**Importance:**

Clinical trials have demonstrated the antifracture efficacy of bisphosphonate drugs for the first 3 to 5 years of therapy. However, the efficacy of continuing bisphosphonate for as long as 10 years is uncertain.

**Objective:**

To examine the association of discontinuing bisphosphonate at study entry, discontinuing at 2 years, and continuing for 5 additional years with the risk of hip fracture among women who had completed 5 years of bisphosphonate treatment at study entry.

**Design, Setting, and Participants:**

This cohort study included women who were members of Kaiser Permanente Northern and Southern California, 2 integrated health care delivery systems, and who had initiated oral bisphosphonate and completed 5 years of treatment by January 1, 2002, to September 30, 2014. Data analysis was conducted from January 2018 to August 2020.

**Exposure:**

Discontinuation of bisphosphonate at study entry (within a 6-month grace period), discontinuation at 2 years (within a 6-month grace period), and continuation for 5 additional years.

**Main Outcomes and Measures:**

The outcome was hip fracture determined by principal hospital discharge diagnoses. Demographic, clinical, and pharmacological data were ascertained from electronic health records.

**Results:**

Among 29 685 women (median [interquartile range] age, 71 [64-77] years; 17 778 [60%] non-Hispanic White individuals), 507 incident hip fractures were identified. Compared with bisphosphonate discontinuation at study entry, there were no differences in the cumulative incidence (ie, risk) of hip fracture if women remained on therapy for 2 additional years (5-year risk difference [RD], −2.2 per 1000 individuals; 95% CI, −20.3 to 15.9 per 1000 individuals) or if women continued therapy for 5 additional years (5-year RD, 3.8 per 1000 individuals; 95% CI, −7.4 to 15.0 per 1000 individuals). While 5-year differences in hip fracture risk comparing continuation for 5 additional years with discontinuation at 2 additional years were not statistically significant (5-year RD, 6.0 per 1000 individuals; 95% CI, −9.9 to 22.0 per 1000 individuals), interim hip fracture risk appeared lower if women discontinued after 2 additional years (3-year RD, 2.8 per 1000 individuals; 95% CI, 1.3 to 4.3 per 1000 individuals; 4-year RD, 9.3 per 1000 individuals; 95% CI, 6.3 to 12.3 per 1000 individuals) but not without a 6-month grace period to define discontinuation.

**Conclusions and Relevance:**

In this study of women treated with bisphosphonate for 5 years, hip fracture risk did not differ if they discontinued treatment compared with continuing treatment for 5 additional years. If women continued for 2 additional years and then discontinued, their risk appeared lower than continuing for 5 additional years. Discontinuation at other times and fracture rates during intervening years should be further studied.

## Key Points

**Question:**

Is bisphosphonate therapy beyond 5 years associated with lower risk of hip fracture?

**Findings:**

In this cohort study of 29 685 older women who completed 5 years of bisphosphonate treatment, if women continued treatment for 5 additional years, the risk of hip fracture was not significantly different than if they discontinued after the first 5 years. If women continued for 2 additional years and then discontinued, there was a difference in hip fracture outcome depending on the use of a grace period for discontinuation.

**Meaning:**

In this study of women who completed 5 years of bisphosphonate treatment, completing an additional 5 years of treatment was not associated with a reduction in hip fracture risk; the potential hip fracture benefit for continuing 2 additional years but not for 5 additional years should be further studied.

## Teaser

This cohort study examines the association of discontinuing bisphosphonate at study entry, discontinuing at 2 years, and continuing for 5 additional years with hip fracture among women who completed 5 years of treatment at study entry.

## Introduction

For more than 2 decades, bisphosphonate drugs have been a first-line therapy for preventing osteoporotic fractures, based on strong clinical trial evidence showing significant fracture risk reduction within the first 3 to 5 years of treatment.^1,2^ However, data regarding the optimal duration of bisphosphonate treatment to minimize fracture risk beyond this initial period are limited.

In September 2015, a Task Force of the American Society Bone and Mineral Research recommended that a drug holiday be considered following 5 years of oral bisphosphonate treatment in women who were not at high fracture risk, whereas ongoing treatment was felt to benefit women at high fracture risk.^3^ Their recommendations were based on collective evidence from clinical trials that each included fewer than 1000 women who continued oral bisphosphonate treatment beyond 5 years.^3^ The task force acknowledged the limited evidence as well as its primary focus on symptomatic vertebral fractures and called for more studies of representative populations.^3^ The US Food and Drug Administration analyzed pooled data^4^ from the extension phase of major bisphosphonate clinical trials^5,6,7^ and concluded that the benefits of continued treatment beyond 3 to 5 years remained unclear. A retrospective Women’s Health Initiative (WHI) study of women who self-reported at least 2 years of bisphosphonate use did not find significant associations of 3 to 5 or 6 to 9 years of treatment with hip fracture, although a 5-year increase in bisphosphonate treatment was associated with increased risk of hip fracture.^8^ These reports emphasize the importance of distinguishing efficacy during the first 5 years of bisphosphonate treatment from efficacy beyond this period.^9^

Difficulty in randomizing patients to treatment cessation or continuation beyond 5 years, the inclusion of sufficient numbers of women, and the long follow-up required are challenges faced by trials seeking to examine the efficacy of bisphosphonate treatment beyond 5 years.^9^ On the other hand, real-world clinical populations can provide as much as 2 decades of experience with oral bisphosphonate drugs, beginning with the market introduction of alendronate in 1995 and risedronate in 1998.^10^ In this study, we used data from Kaiser Permanente Northern and Southern California, the 2 largest integrated health care delivery systems within the western United States, whose centralized databases have tracked both oral bisphosphonate exposure and hip fracture events for more than 2 decades.^11,12,13,14,15,16^ Our analytic approach uses observational data to emulate the results of a 5-year trial to examine the association of bisphosphonate discontinuation at study entry (total exposure, 5 years), discontinuation 2 years later (total exposure, 7 years), and continuation for 5 additional years (total exposure, 10 years) with the risk of hip fracture in older women who completed 5 years of treatment.

## Methods

### Study Population

This retrospective study used data from Kaiser Permanente Northern (KPNC) and Southern California (KPSC) to identify women who initiated oral bisphosphonate therapy with alendronate, risedronate, or ibandronate between January 1, 1997, and September 30, 2009; were aged 45 to 80 years at bisphosphonate initiation; had health plan membership during the 2 prior years; and received at least 60% adherent bisphosphonate treatment in each of the 5 years after initiation. Drug adherence was based on the days’ supply of dispensed prescriptions. Study follow-up commenced between 2002 to 2014, after the first 5 years of treatment, referred to as the *index date*. Women were also required to have received bisphosphonate treatment within 60 days prior to the index date. Women were excluded if they previously received intravenous bisphosphonate or etidronate; received denosumab, teriparatide, raloxifene, or estrogen in the 2 years prior to the index date; had advanced (grade 4-5) or end-stage kidney disease; or were diagnosed with a metabolic bone condition (Paget disease, osteogenesis imperfecta, hypophosphatasia, or primary hyperparathyroidism), secondary or metastatic cancer, or multiple myeloma, as previously described.^17,18^ Participants were followed up from their index date until the earliest occurrence of a hip fracture (n = 507), death (n = 1164), an exclusionary event (n = 1676), end of membership (n = 1958), or study end 5 years after index date (n = 24 353).

The study was approved by the KPNC and KPSC institutional review boards, and waivers of informed consent were obtained due to the nature of the study. This report follows the Strengthening the Reporting of Observational Studies in Epidemiology (STROBE) reporting guideline.

### Bisphosphonate Exposure Assessment

Following the index date and for each successive 90-day interval (quarter) of follow-up, women were categorized as receiving bisphosphonate if at least 60% of the time period was covered by days’ supply of drug in health plan pharmacy records (proportion of days covered [PDC], ≥0.60).^17,18^ Women were considered not receiving bisphosphonate if they received drug for less than the specified PDC. Stockpiling was allowed for bisphosphonate prescriptions that overlapped by 30 days or less. When prescriptions overlapped for more than 30 days, the second prescription took precedence.^19^ Classification of the 3 bisphosphonate regimens examined is described in Statistical Analysis section.

### Hip Fracture Assessment

The primary end point was proximal femur (hip) fracture, defined by a principal hospital discharge diagnosis of closed fracture of the femur (transcervical, pertrochanter, or femoral neck, *International Classification of Diseases, Ninth Revision *[*ICD-9*] codes 820.0x, 820.2x, or 820.8x), excluding open fractures codes and those associated with major trauma (*ICD-9* codes E800-E848).

### Covariates

We considered an extensive set of baseline and time-dependent covariates that may be associated with treatment^17^ or the risk of hip fracture. Baseline measures included age (in 5-year increments) and self-reported race/ethnicity (classified as non-Hispanic White, Hispanic/Latina, Black, Asian or Pacific Islander, and other or unknown). We used US Census data to estimate surrogate markers of socioeconomic status, including low educational attainment (residence in a census block where >25% of individuals aged >25 years had less than a 12th grade education) and low income (residence in a census block with a median annual household income <$35 000).^17^ Body mass index (calculated as weight in kilograms divided by height in meters squared and categorized as normal/underweight [<25], overweight [25 to <30], or obesity [≥30]), self-reported smoking status, evidence of vitamin D deficiency (25-hydroxyvitamin D level [25-OHD] <20 ng/mL [to convert to picomoles per liter, multiply by 2.496]), and an indicator of 25-OHD assessment were obtained within 5 years of the index date. We additionally considered indicators of hip, humerus, vertebral, wrist, or other fracture^17^ prior to bisphosphonate initiation. An indicator of whether a woman experienced a hip fracture in the 5 years between bisphosphonate initiation and the index date was included in analyses. Participants were classified by region (KPNC or KPSC) and cohort entry before or after 2008, the year in which revised osteoporosis guidelines were published^20^ and the fracture risk assessment tool (FRAX) was introduced.^21,22^

The set of time-dependent covariates included the most recent Charlson-Deyo Comorbidity Index^23^ derived from diagnosis and procedure codes obtained from encounters in the prior year; evidence of diabetes (≥2 diagnoses and pharmacologic treatment); rheumatoid arthritis (≥2 diagnoses); grade 3A or 3B chronic kidney disease (outpatient estimated glomerular filtration rate [eGFR], 45-59 mL/min/1.7 m^2^ and 30-44 mL/min/1.7 m^2^, respectively); indicators of spine, humerus, wrist, or other clinical fracture^17^ within the prior 5 years for baseline and the prior 12 months during follow-up; most recent bone mineral density (BMD) T score (described in next paragraph); and treatment with proton-pump inhibitors, aromatase inhibitors, or systemic oral glucocorticoids (cumulative prednisone dose equivalent ≥1825 mg received in the prior year, averaging 5 mg/d). For participants with missing eGFR at baseline (7742 [26%]) the mean baseline value (74 mL/min/1.7 m^2^) was imputed, and an indicator of whether the value was imputed was included in analyses.^24,25,26^ The last observed value was carried forward (LOVCF) to subsequent quarters, and the indicator of LOVCF was included in the covariate adjustment set.^27^

BMD measurements up to 5 years before the index date and during follow-up included femoral neck, total hip, and lumbar spine BMD and T scores^28^ from Hologic (KPNC) and GE Lunar (KPSC) bone densitometers. The lowest BMD T score (from measured sites for each BMD test) was used in analyses and categorized as greater than −2.0, −2.0 to −2.4, −2.5 to −2.9, and−3.0 or less. The mean BMD T score was imputed for participants with missing BMD at baseline (18 156 [61%]), and an indicator of whether T score was imputed was included in analyses.

### Statistical Analysis

Our analytic approach used observational data to emulate the results^29^ of a 5-year trial in which women in the study cohort would have been randomized at their index date to 1 of 3 exposure regimens of interest: regimen 1, discontinuation of bisphosphonate therapy and continuous absence of adherent treatment thereafter (ie, total 5 years of bisphosphonate use); regimen 2, discontinuation of bisphosphonate therapy at the end of 2 additional years of treatment and continuous absence of adherent treatment thereafter (ie, total 7 consecutive years of bisphosphonate use); and regimen 3, continuous bisphosphonate therapy for 5 additional years (ie, total 10 years of bisphosphonate use). For the first 2 exposure groups, the emulated trial protocol additionally allowed a 6-month window (ie, grace period)^30^ for bisphosphonate discontinuation to occur. That is, discontinuation could occur in the first 6 months after the index date for the first regimen, and in the six months following 2 additional years of treatment after the index date for the second regimen.

To estimate the three 5-year survival curves and corresponding cumulative risk differences (RDs) between any 2 groups of the emulated trial, we used inverse probability weighting (IPW),^31,32^ a propensity score approach, instead of standard statistical methods to properly adjust for complex time-varying confounding. Standard analytic methods (eg, Cox regression or propensity score matching) cannot properly handle time-varying confounding by a factor that is affected by past exposure status and is associated with both future exposure status and subsequent outcomes. Separate multivariable logistic regression models were fit to estimate propensity scores, which include the probability of bisphosphonate initiation and continuation as well as the probability of each of the 4 censoring events (ie, death, disenrollment, study end, and exclusionary events). Models included all baseline and last-observed time-dependent covariates as described earlier. In addition, we adjusted for cumulative bisphosphonate exposure, for length of follow-up, and for the time since the most recent BMD test, given that recent BMD results may be a more important determinant of treatment continuation than historical measurements. IPWs were stabilized^33,34^ and truncated at 50; truncation of stabilized weights affected less than 2% of observations. Distributions of stabilized weights are provided in eTables 1, 2, 3, 9, and 10 in the Supplement.

In addition to our main IPW analyses, we computed effect estimates using targeted minimum loss-based estimation (TMLE),^35^ an approach that can also properly adjust for time-varying confounding and additionally has desirable statistical properties (eg, more precise effect estimates) discussed in the eAppendix in the Supplement. Distributions of unstabilized weights used by TMLE are provided in eTables 5, 6, 7, 12, and 13 in the Supplement. Analytic data sets were created in SAS version 9.4 (SAS Institute) using the MSMStructure SAS Macro.^36^ Measurements on exposure, outcome (incident hip fracture), censoring, and time-dependent covariates were updated every quarter between the index date until the end of follow-up. Analyses were performed using the stremr package^37^ in R version 3.4.4 (R Project for Statistical Computing). The level of significance was set at *P* < .05, and all tests were 2-tailed.

We note that our analytic approach did not make arbitrary assumptions such as the proportionality assumption, ie, hazards ratios were permitted to change over time. Thus, we report adjusted survival curves and cumulative RDs as the primary effect measures as opposed to hazard ratios.^38^

## Results

Demographic and clinical characteristics of our study population are presented in Table 1. Our study included 29 685 women who had completed 5 initial years of bisphosphonate treatment at the start of follow-up (index date) with a median (interquartile range) age of 71 (64-77) years; 17 778 (60%) were non-Hispanic White individuals, 3785 (13%) were Hispanic/Latina individuals, 6045 (20%) were Asian or Pacific Islander individuals, and 1281 (4%) were Black individuals. Overall, 666 participants (2%) had experienced a hip fracture; 3384 (11%), humerus, spine, or wrist fracture; and 8224 (28%), any clinical fracture. Among those with available BMD measurements prior to the index date, 4291 (37%) had osteoporosis. Among all women who entered the cohort, 11 105 (37%) continued bisphosphonate treatment for 2 additional years, for a total of 7 years of treatment, and 2725 (9.2%) remained on treatment for all 5 follow-up years, completing a total of 10 years of treatment. The numbers of regimen followers in each quarter of follow-up are provided in eFigure 1 and eFigure 5 in the Supplement.

**Table 1.** Baseline Characteristics of Women Who Received 5 Years of Bisphosphonate Therapy

Characteristic	No. (%) (N = 29 685)
Location	
Northern California	12 391 (42)
Southern California	17 294 (58)
Age, median (IQR), y	71 (64-77)
Race/ethnicity	
Non-Hispanic White	17 778 (60)
African-American or Black	1281 (4)
Hispanic or Latina	3785 (13)
Asian or Pacific Islander	6045 (20)
Other, mixed race, or unknown	796 (3)
Index BMI category	
Normal or underweight, <25	16 621 (56)
Overweight, 25 to <30	9149 (31)
Obesity, ≥30	4035 (14)
Unknown	585 (2)
Current smoking	1942 (6)
Estimated low education attainment	4543 (15)
Estimated low household income	1884 (6)
Charlson Comorbidity Index score	
0	18 319 (62)
1-2	8612 (29)
>2	2754 (9)
History of medical conditions	
Diabetes	2367 (8)
Rheumatoid arthritis	962 (3)
Grade 3 chronic kidney disease	4744 (16)
Vitamin D deficiency	3329 (11)
Fracture history prior to index date	
Hip	666 (2)
Other major osteoporotic^a^	3384 (11)
Any clinical fracture	8224 (28)
Bone mineral density	
DXA tested	11 529 (39)
Osteoporosis^b^	4291 (37)
Osteopenia^b^	6547 (57)

Abbreviations: BMI, body mass index (calculated as weight in kilograms divided by height in meters squared); DXA, dual-energy x-ray absorptiometry; IQR, interquartile range.

^a^ Includes fractures of the humerus, wrist, or spine.

^b^ Osteoporosis was indicated by a T score of −2.5 or less; osteopenia by a T score between −2.5 and −1.

**Figure 1.**
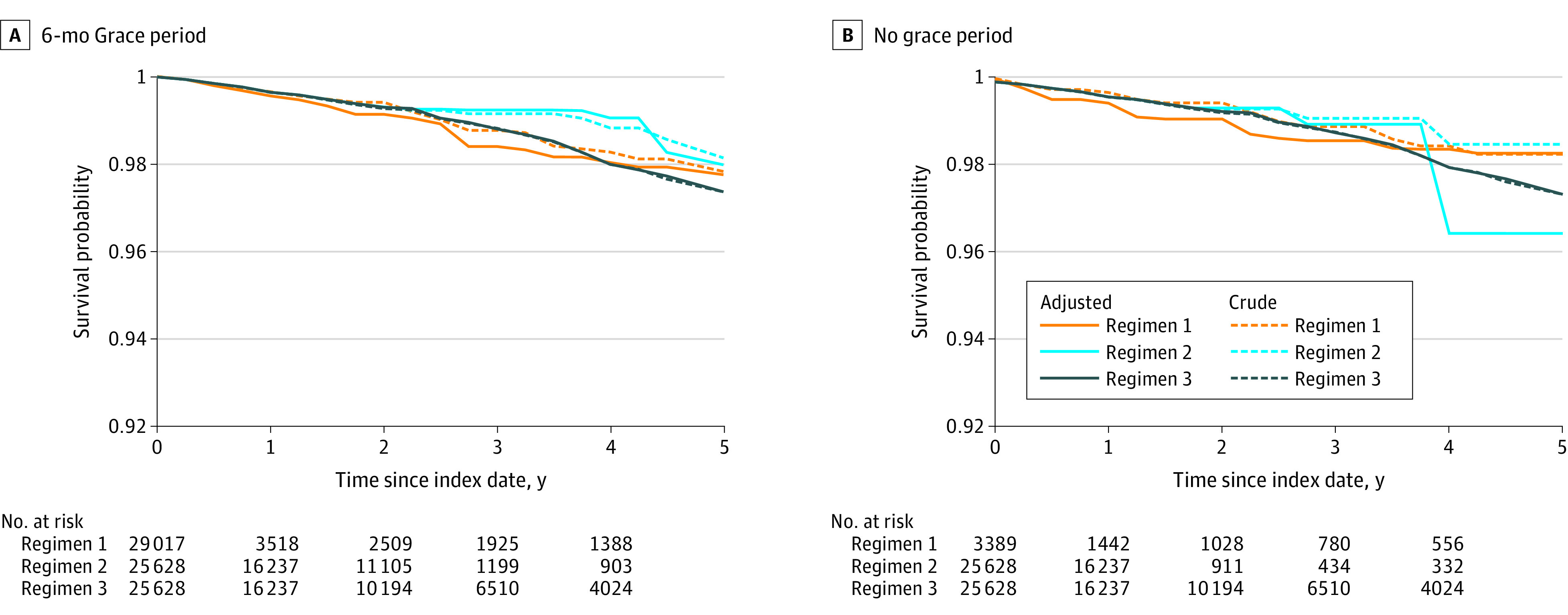
Hip Fracture Survival Regimen 1 indicates discontinuation of bisphosphonate at study entry; regimen 2, discontinuation after 2 additional years of treatment; regimen 3, continuation of bisphosphonate treatment for all 5 years of follow-up.

We identified 507 incident hip fractures during the 5 years of follow-up after study entry, including 292 (58%) and 251 (50%) that occurred while women were following 1 of the regimens, with and without a 6-month grace period, respectively. Crude and adjusted survival curves showed no differences in hip fracture–free survival between continuing bisphosphonate therapy for 5 additional years vs discontinuing bisphosphonate at study entry (Figure 1; eFigures 2, 3, 6, and 7 in the Supplement). Adjusted survival curves suggested better hip fracture–free survival if women interrupted bisphosphonate at 2 additional years compared with either discontinuing at study entry or continuing for 5 additional years, but these differences were attenuated when the 6-month grace period for bisphosphonate discontinuation was not included (Figure 1; eFigures 2, 3, 6, and 7 in the Supplement). The TMLE survival curves followed a similar pattern (eFigure 4 and eFigure 8 in the Supplement).

**Figure 2.**
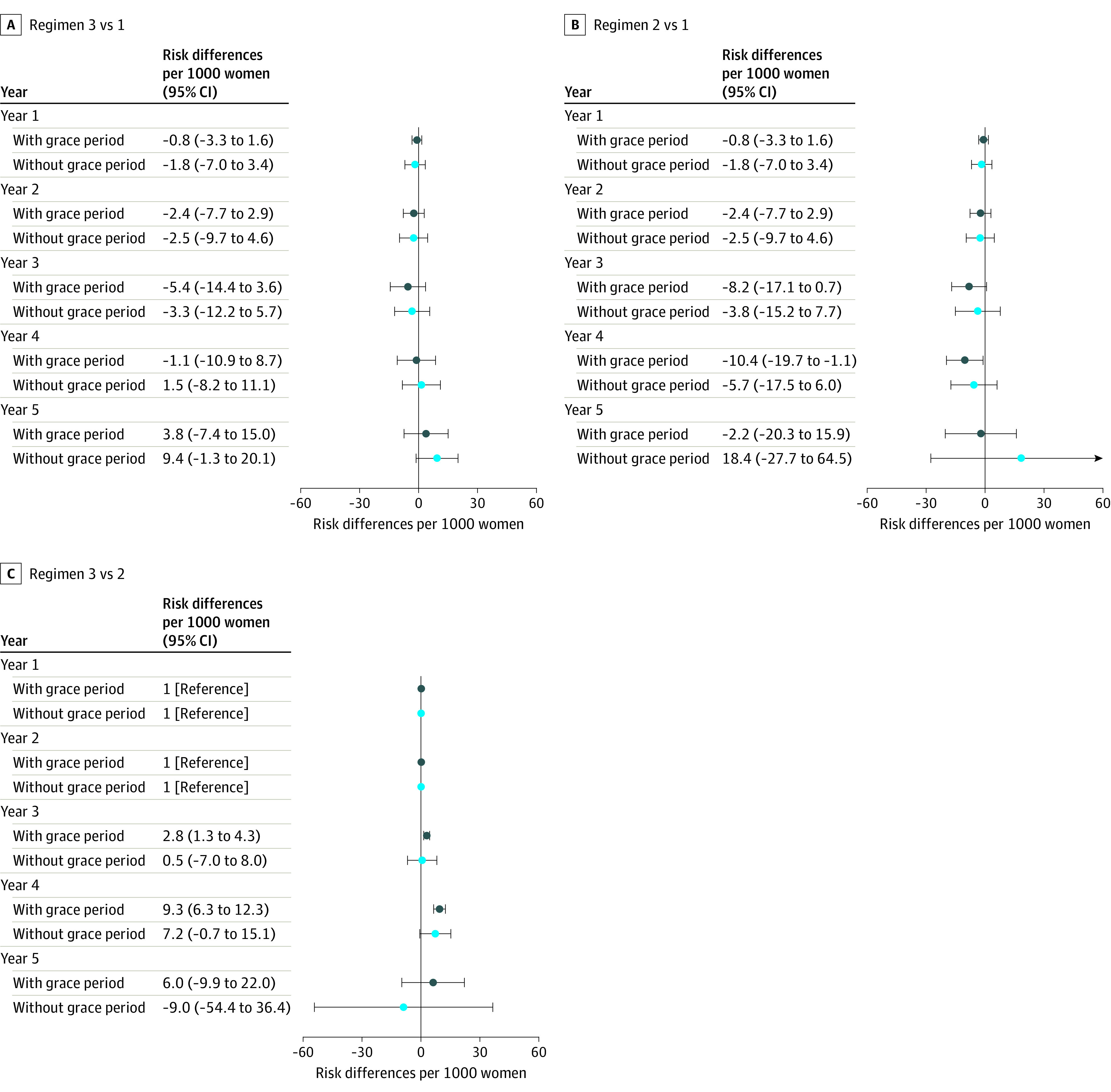
Adjusted Risk Differences for Hip Fracture Regimen 1 indicates discontinuation of bisphosphonate at study entry; regimen 2, discontinuation after 2 additional years of treatment; regimen 3, continuation of bisphosphonate treatment for all 5 years of follow-up.

The 5-year cumulative incidences (risks) of hip fracture were 23.0 and 20.8 per 1000 individuals if women stopped taking bisphosphonate at study entry or after 2 additional years of treatment when allowing a 6-month grace period (Table 2). The 5-year risk of hip fracture was 26.8 per 1000 individuals if women remained on bisphosphonate for all 5 years. There were no statistically significant differences between continuing bisphosphonate for 5 additional years and discontinuing at study entry with a 6-month grace period (5-year RD, 3.8 per 1000 individuals; 95% CI, −7.4 to 15.0 per 1000 individuals) (Figure 2; eTable 4 in the Supplement) or with no grace period (RD, 9.4 per 1000 individuals; 95% CI, −1.3 to 20.1 per 1000 individuals) (eTable 11 in the Supplement). Additionally, there were no significant differences in the 5-year hip fracture risk if women discontinued treatment after 2 additional years compared with discontinuation of treatment at study entry (with a 6-month grace period: 5-year RD, −2.2 per 1000 individuals; 95% CI, −20.3 to 15.9 per 1000 individuals; with no grace period: 5-year RD, 18.4 per 1000 individuals; 95% CI, −27.7 to 64.5 per 1000 individuals), although a statistically significant decrease in the 4-year risk was seen if women continued for 2 additional years compared with discontinuing at entry (4-year RD, −10.4 per 1000 individuals; 95% CI, −19.7 to −1.1 per 1000 individuals), but only with the inclusion of a 6-month grace period. We did not observe significant differences in the 5-year risk of hip fracture if women continued bisphosphonate treatment for all 5 years compared with interrupting after 2 years (with a grace period: 5-year RD, 6.0 per 1000 individuals; 95% CI, −9.9 to 22.0 per 1000 individuals; without a grace period: RD, −9.0 per 1000 individuals; 95% CI, −54.4 to 36.4 per 1000 individuals), although statistically significant increases in the risk of hip fracture with continued treatment were seen in years 3 (RD, 2.8 per 1000 individuals; 95% CI, 1.3 to 4.3 per 1000 individuals) and 4 (RD, 9.3 per 1000 individuals; 95% CI, 6.3 to 12.3 per 1000 individuals) compared with discontinuing at 2 years, again only with inclusion of a 6-month grace period (Figure 2). The TMLE results followed a similar pattern (eTable 8 and eTable 14 in the Supplement).

**Table 2.** Adjusted Cumulative Incidence of Hip Fracture at the End of Each Follow-up Year, With and Without a Grace Period

Year	Regimen 1, discontinuation at study entry				Regimen 2, discontinuation at 2 y				Regimen 3, continuation for 5 y	
	6-mo Grace period		No grace period		6-mo Grace period		No grace period			
	Cumulative incidence per 1000 individuals	Events per year, No.	Cumulative incidence per 1000 individuals	Events per year, No.	Cumulative incidence per 1000 individuals	Events per year, No.	Cumulative incidence per 1000 individuals	Events per year, No.	Cumulative incidence per 1000 individuals	Events per year. No.
1	4.2 (1.7-6.6)	56	5.1 (0.0-10.2)	6	3.3 (2.6-4.1)	74	3.3 (2.6-4.1)	74	3.3 (2.6-4.1)	74
2	9.5 (4.3-14.7)	10	9.6 (2.5-16.6)	4	7.0 (5.8-8.3)	54	7.0 (5.8-8.3)	54	7.0 (5.8-8.3)	54
3	16.7 (7.9-25.5)	14	14.5 (5.8-23.3)	5	8.5 (7.0-10.0)	15	10.8 (3.4-18.2)	1	11.3 (9.3-13.3)	36
4	19.1 (9.8-28.3)	7	16.5 (7.4-25.6)	3	8.6 (7.1-10.2)	1	10.8 (3.4-18.2)	0	18.0 (14.7-21.2)	35
5	23.0 (13.1-32.9)	6	17.4 (8.0-26.7)	1	20.8 (5.6-36.0)	7	35.8 (0.0-80.9)	2	26.8 (21.6-32.1)	30

## Discussion

In a large cohort of older women who completed 5 initial years of bisphosphonate treatment, we found a similar 5-year risk of hip fracture whether women discontinued bisphosphonate treatment at study entry or continued adherent treatment for 5 additional years. These findings were evident in analyses when defining discontinuation with or without a 6-month grace period. For comparisons of hip fracture risk if women continued bisphosphonate treatment for 2 additional years, findings were mixed, depending on use of a 6-month grace period for discontinuation, which allowed the inclusion of a larger number of women. While our results suggest that interruption of bisphosphonate treatment after approximately 2 additional treatment years may be associated with lower interim risk of hip fracture compared with women who continued bisphosphonate treatment for 5 additional years, the 5-year risk differences were not statistically significant and the numbers of women and hip fracture events in this exposure group were relatively small.

The Fracture Intervention Trials (FIT)^39,40^ and Vertebral Efficacy with Risedronate Therapy (VERT) trials^41,42^ demonstrated that bisphosphonate therapy reduced the risk of hip, vertebral, and nonvertebral clinical fractures in women with osteoporosis during the first 3 to 5 years. However, few trials to our knowledge have examined bisphosphonate efficacy beyond 5 years. A 5-year FIT trial extension (FLEX) conducted among 1099 women examined the efficacy of continuing or stopping alendronate after 3 to 5 years and found that women who stopped experienced no greater overall fracture risk for most clinical fractures compared with those continuining alendronate to 10 years, except for clinical vertebral fracture risk reduction but not those measured radiographically.^6^ In post hoc subgroup analyses, nonvertebral fracture risk reduction occurred in those without existing vertebral fractures who had hip osteoporosis by BMD, but there was no significant benefit to longer-term alendronate in the highest risk group (ie, those with prior vertebral fractures and low BMD).^6,43^ Some felt that these findings and 4- to 7-year risedronate extension studies^7^ suggested that bisphosphonate continuation beyond 3 to 5 years may afford fracture protection in higher risk patients with osteoporosis, but existing trials examined fracture outcomes in relatively small numbers of women with more than 5 years of bisphosphonate therapy and were not able to examine hip fractures.

Our observational study was conducted in a large cohort of women previously exposed to long-term bisphosphonate treatment. Other observational studies evaluating long-term bisphosphonate treatment on hip fracture risk reported mixed findings. A small study of 183 women who received 3 to 5 years of bisphosphonate reported increased risk of new clinical fractures among patients who discontinued treatment.^44^ Another study examining 81 427 Medicare beneficiaries with at least 3 years of bisphosphonate use (80% adherence; most <5 years) reported increased hip fracture risk among women who discontinued treatment for more than 2 years.^45^ A nested case-control study in Denmark (drawn from 61 990 adults; 51 558 women) found that bisphosphonate treatment for 5 to 10 years and more than 10 years were both associated with 30% lower hip fracture risk compared with less than 5 years treatment.^46^ In contrast, the WHI study of 5120 women with at least 2 years of bisphosphonate use found that each 5-year increase in use was associated with a statistically significant increase in hip fracture risk (adjusted hazard ratio, 1.33).^8^ A recent retrospective study of women with at least 3 years bisphosphonate therapy showed no increased hip fracture risk among those given a bisphosphonate holiday of at least 12 months.^16^ In our study, continuing bisphosphonate for 5 additional years did not appear to result in hip fracture benefit. However, whether there is hip fracture benefit from continuing bisphosphonate treatment for only 2 additional years should be further studied.

### Strengths and Limitations

This study has several strengths. A main strength is the inclusion of a large and diverse population of older women who already had long-term bisphosphonate use. Centralized electronic health databases provided an accurate assessment of bisphosphonate exposure, hip fracture events, and a comprehensive set of time-dependent potential confounders. We used statistical methods that accounted for numerous potential factors that could be simultaneously associated with both treatment continuation and hip fracture risk.

This study has limitations. First, we used observational data that required some imputation (including missing BMD), but indicators of missing data were used in our analyses.^24,25^ Second, these findings may not be applicable to older women at higher risk of osteoporotic fracture. Third, we used an adherence threshold of 60% to reflect real-world treatment behaviors, although higher^47^ and lower^16^ thresholds have been used in studies examining fracture risk. We did not address the optimal duration of bisphosphonate treatment or effects of temporary interruption. Fourth, we cannot exclude the possibility that the outcome included atypical femur fracture, which has very low incidence compared with hip fracture, but femoral shaft–coded fractures (a frequent site of atypical fracture) were not included. Finally, our analytic approach used propensity scores to account for differences between treatment arms, but as in any observational study, residual unmeasured confounding is possible. Propensity scores were estimated using parametric regression models rather than more flexible machine learning approaches, which could result in residual confounding by observed covariates.^48^

## Conclusions

In this cohort study of older women who already received 5 years of bisphosphonate therapy, there appeared to be neither hip fracture benefit nor harm associated with discontinuing treatment at study entry or continuing treatment for an additional 5 years. A potential benefit was suggested from continuing an additional 2 years, but the association of discontinuation at 2 years and other points with fracture risk should be further studied in randomized clinical trials. Future investigation should also examine whether such findings apply to women at higher or lower fracture risk and to what degree other fracture outcomes might be affected. Our findings of hip fracture risk are similar to the FLEX randomized clinical trial, in which overall clinical fracture risk was not significantly different between those who took placebo or alendronate for an additional 5 years.^6^

## Notes

Supplement. **eAppendix. **Brief Notes on the TMLE **eFigure 1. **Number of Women Who Continue to Follow, Are No Longer Following, or Were Censored from Regimens 1, 2, and 3 (With Grace Period) **eFigure 2. **Crude Survival Probabilities for the 3 Regimens of Interest (With Grace Period) **eTable 1. **Distribution of Stabilized Weights Among Regimen 1 Followers, IPW (With Grace Period) **eTable 2. **Distribution of Stabilized Weights Among Regimen 2 Followers, IPW (With Grace Period) **eTable 3. **Distribution of Stabilized Weights Among Regimen 3 Followers, IPW (With Grace Period) **eFigure 3. **IPW Survival Probabilities for the 3 Regimens of Interest (With Grace Period) **eTable 4. **IPW Estimated Risk Differences per 1000 Women Comparing Regimens of Interest (With Grace Period) **eTable 5. **Distribution of Unstabilized Weights Among Regimen 1 Followers, TMLE (With Grace Period) **eTable 6. **Distribution of Unstabilized Weights Among Regimen 2 Followers, TMLE (With Grace Period) **eTable 7. **Distribution of Unstabilized Weights Among Regimen 3 Followers, TMLE (With Grace Period) **eFigure 4. **TMLE Survival Probabilities for the 3 Regimens of Interest (With Grace Period) **eTable 8. **TMLE Risk Differences per 1000 Women Comparing Regimens of Interest (With Grace Period) **eFigure 5. **Number of Women Who Continue to Follow, Are No Longer Following, or Were Censored from Regimens 1, 2, and 3 (Without Grace Period) **eFigure 6. **Crude Survival Probabilities for the 3 Regimens of Interest (Without Grace Period) **eTable 9. **Distribution of Stabilized Weights Among Regimen 1 Followers, IPW (Without Grace Period) **eTable 10. **Distribution of Stabilized Weights Among Regimen 2 Followers, IPW (Without Grace Period) **eFigure 7. **IPW Survival Probabilities for the 3 Regimens of Interest (Without Grace Period) **eTable 11. **IPW Estimated Risk Differences per 1000 Women Comparing Regimens of Interest (Without Grace Period) **eTable 12. **Distribution of Unstabilized Weights Among Regimen 1 Followers, TMLE (Without Grace Period) **eTable 13. **Distribution of Unstabilized Weights Among Regimen 2 Followers, TMLE (Without Grace Period) **eFigure 8. **TMLE Survival Probabilities for the 3 Regimens of Interest (Without Grace Period) **eTable 14. **TMLE Risk Differences per 1000 Women Comparing Regimens of Interest (Without Grace Period)
