# Supplementary material for: Bisphosphonate Treatment Beyond 5 Years and Hip Fracture Risk in Older Women
Source: JAMA Netw Open. 2020 Dec 7;3(12):e2025190. doi: 10.1001/jamanetworkopen.2020.25190 (PMC8436954; doi:10.1001/jamanetworkopen.2020.25190)
Supplement: Supplement. — eAppendix. Brief Notes on the TMLE eFigure 1. Number of Women Who Continue to Follow, Are No Longer Following, or Were Censored from Regimens 1, 2, and 3 (With Grace Period) eFigure 2. Crude Survival Probabilities for the 3 Regimens of Interest (With Grace Period) eTable 1. Distribution of Stabilized Weights Among Regimen 1 Followers, IPW (With Grace Period) eTable 2. Distribution of Stabilized Weights Among Regimen 2 Followers, IPW (With Grace Period) eTable 3. Distribution of Stabilized Weights Among Regimen 3 Followers, IPW (With Grace Period) eFigure 3. IPW Survival Probabilities for the 3 Regimens of Interest (With Grace Period) eTable 4. IPW Estimated Risk Differences per 1000 Women Comparing Regimens of Interest (With Grace Period) eTable 5. Distribution of Unstabilized Weights Among Regimen 1 Followers, TMLE (With Grace Period) eTable 6. Distribution of Unstabilized Weights Among Regimen 2 Followers, TMLE (With Grace Period) eTable 7. Distribution of Unstabilized Weights Among Regimen 3 Followers, TMLE (With Grace Period) eFigure 4. TMLE Survival Probabilities for the 3 Regimens of Interest (With Grace Period) eTable 8. TMLE Risk Differences per 1000 Women Comparing Regimens of Interest (With Grace Period) eFigure 5. Number of Women Who Continue to Follow, Are No Longer Following, or Were Censored from Regimens 1, 2, and 3 (Without Grace Period) eFigure 6. Crude Survival Probabilities for the 3 Regimens of Interest (Without Grace Period) eTable 9. Distribution of Stabilized Weights Among Regimen 1 Followers, IPW (Without Grace Period) eTable 10. Distribution of Stabilized Weights Among Regimen 2 Followers, IPW (Without Grace Period) eFigure 7. IPW Survival Probabilities for the 3 Regimens of Interest (Without Grace Period) eTable 11. IPW Estimated Risk Differences per 1000 Women Comparing Regimens of Interest (Without Grace Period) eTable 12. Distribution of Unstabilized Weights Among Regimen 1 Followers, TMLE (Without Grace Period) eTable 13. Distribution o [file jamanetwopen-e2025190-s001.pdf]

Supplemental Online Content

Izano MA, Lo JC, Adams AL, et al. Bisphosphonate treatment beyond 5 years and hip fracture risk in older women. *JAMA Netw Open*. 2020;3(12):e2025190.
doi:10.1001/jamanetworkopen.2020.25190

eAppendix. Brief Notes on the TMLE
eFigure 1. Number of Women Who Continue to Follow, Are No Longer Following, or Were Censored from Regimens 1, 2, and 3 (With Grace Period)
eFigure 2. Crude Survival Probabilities for the 3 Regimens of Interest (With Grace Period)
eTable 1. Distribution of Stabilized Weights Among Regimen 1 Followers, IPW (With Grace Period)
eTable 2. Distribution of Stabilized Weights Among Regimen 2 Followers, IPW (With Grace Period)
eTable 3. Distribution of Stabilized Weights Among Regimen 3 Followers, IPW (With Grace Period)
eFigure 3. IPW Survival Probabilities for the 3 Regimens of Interest (With Grace Period)
eTable 4. IPW Estimated Risk Differences per 1000 Women Comparing Regimens of Interest (With Grace Period)
eTable 5. Distribution of Unstabilized Weights Among Regimen 1 Followers, TMLE (With Grace Period)
eTable 6. Distribution of Unstabilized Weights Among Regimen 2 Followers, TMLE (With Grace Period)
eTable 7. Distribution of Unstabilized Weights Among Regimen 3 Followers, TMLE (With Grace Period)
eFigure 4. TMLE Survival Probabilities for the 3 Regimens of Interest (With Grace Period)
eTable 8. TMLE Risk Differences per 1000 Women Comparing Regimens of Interest (With Grace Period)
eFigure 5. Number of Women Who Continue to Follow, Are No Longer Following, or Were Censored from Regimens 1, 2, and 3 (Without Grace Period)
eFigure 6. Crude Survival Probabilities for the 3 Regimens of Interest (Without Grace Period)
eTable 9. Distribution of Stabilized Weights Among Regimen 1 Followers, IPW (Without Grace Period)
eTable 10. Distribution of Stabilized Weights Among Regimen 2 Followers, IPW (Without Grace Period)
eFigure 7. IPW Survival Probabilities for the 3 Regimens of Interest (Without Grace Period)
eTable 11. IPW Estimated Risk Differences per 1000 Women Comparing Regimens of Interest (Without Grace Period)
eTable 12. Distribution of Unstabilized Weights Among Regimen 1 Followers, TMLE (Without Grace Period)
eTable 13. Distribution of Unstabilized Weights Among Regimen 2 Followers, TMLE (Without Grace Period)
eFigure 8. TMLE Survival Probabilities for the 3 Regimens of Interest (Without Grace Period)
eTable 14. TMLE Risk Differences per 1000 Women Comparing Regimens of Interest (Without Grace Period)

© 2020 Izano MA et al. *JAMA Network Open.*

This supplemental material has been provided by the authors to give readers additional information about their work.

© 2020 Izano MA et al. *JAMA Network Open.*

eAppendix. Brief Notes on the TMLE

Briefly, the TMLE involves estimation of two components: (1) the probability of being exposed (exposure mechanism) and remaining uncensored (censoring mechanism) conditional on covariates (propensity scores), and (2) averages of outcomes conditional on exposure and covariates (outcome models). TMLE can properly adjust for time-varying confounding and informative censoring even if the model for one of the two components (propensity scores or outcome models) is mis-specified (double robustness property). TMLE is efficient when the models for both components are correctly specified.

The exposure, censoring, and outcome models were estimated using multivariable logistic regression. Exposure models were fit separately for the first follow-up quarter; for the subsequent quarters, two separate models were fit among women exposed and unexposed in the previous quarter. Additionally, we fit separate models predicting the probability of censoring by death, health plan disenrollment, study end, and experiencing one of the exclusionary events. Exposure, outcome, and censoring models included all the baseline and time-dependent covariates previously described. Models were additionally adjusted for length of follow-up (less than 6 months, 6 months 1 year, 1-2 years, 2-3 years, 3-4 years, 4-5 years) and the time since most recent BMD testing (6 months or less, 6 months to 2 years, 2-4 years, 4-6 years, more than 6 years) since recent BMD assessments may be a more important determinants of treatment continuation than older ones. All three exposure models were additionally adjusted for cumulative bisphosphonate exposure in the previous quarter of follow-up. For each quarter of follow-up, model-based predicted exposure and censoring probabilities were used to estimate weights defined by inverse propensity scores of remaining uncensored and following the exposure regiments of interest. Since the TMLE uses unstabilized weights, for the TMLE analyses weights were truncated at 100. This number is the ratio of 50, the truncation level for stabilized weights in IPW analyses, and the median numerator of the stabilized weights across the three regimens.

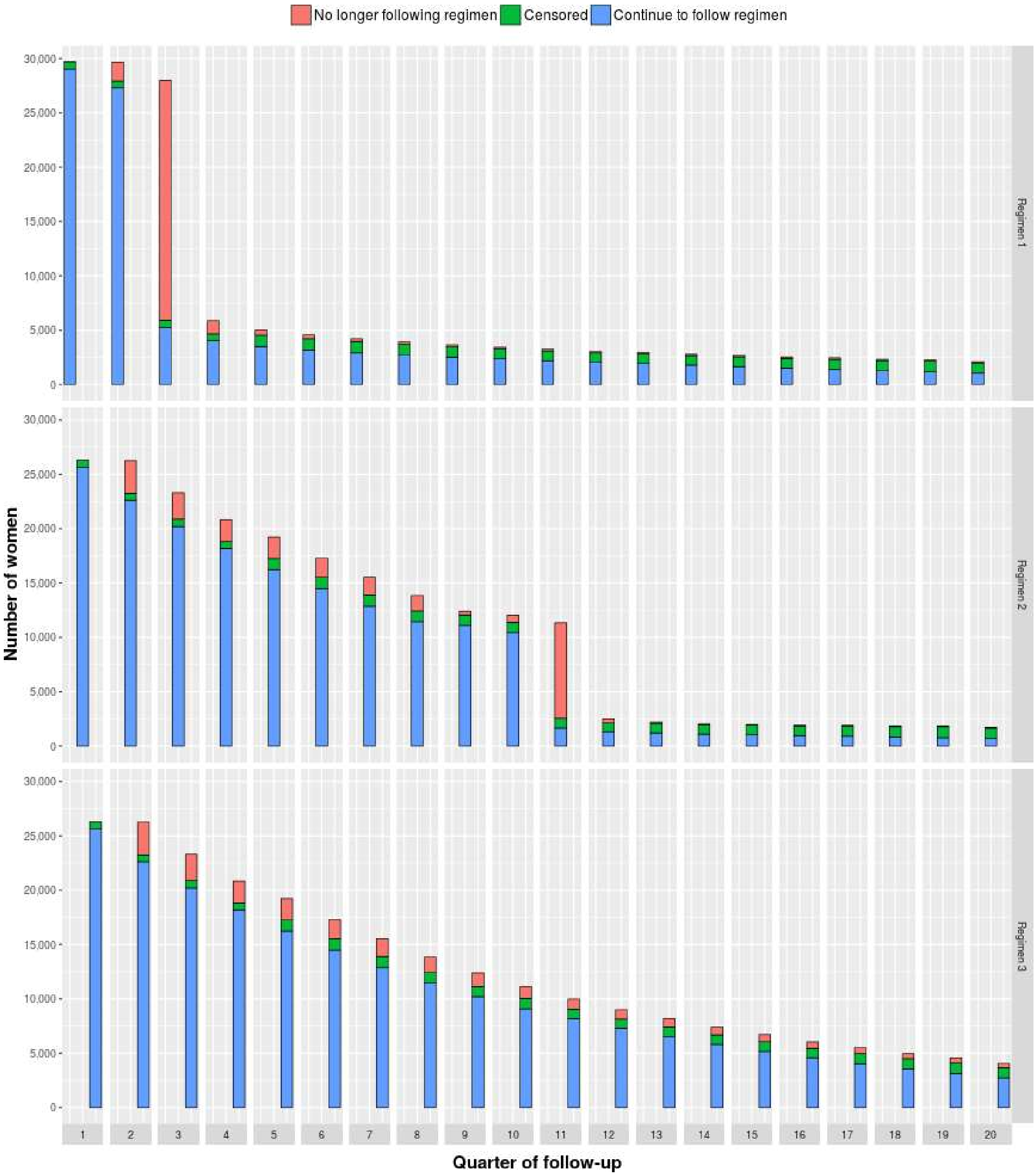

eFigure 1. Number of Women Who Continue to Follow, Are No Longer Following, or Were Censored from Regimens 1, 2, and 3 (With Grace Period)

© 2020 Izano MA et al. *JAMA Network Open.*

Crude Survival Curves

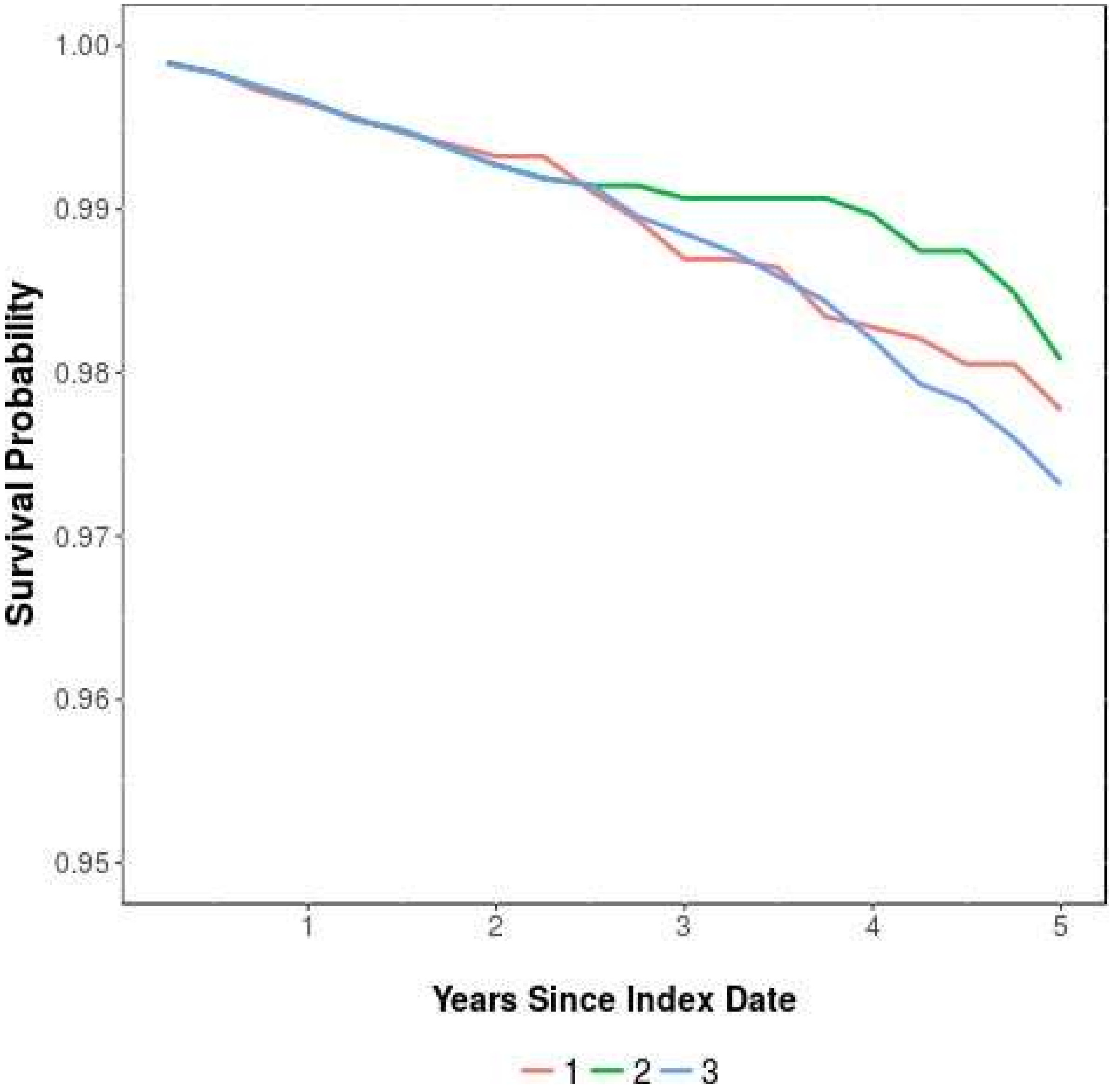

eFigure 2. Crude Survival Probabilities for the 3 Regimens of Interest (With Grace Period) Regimen 1: discontinuation of BP at study entry; Regimen 2: discontinuation of BP at two years; Regimen 3: continuous BP exposure for 5 years.

© 2020 Izano MA et al. *JAMA Network Open.*

eTable 1. Distribution of Stabilized Weights Among Regimen 1 Followers, IPW (With Grace Period)

cum.IPAW	Frequency	%	Cumulative Frequency	Cumulative %
<0	0	0.00	0	0.00
[0, 0.5[	26882	27.22	26882	27.22
[0.5, 1[	46581	47.16	73463	74.38
[1, 10[	24892	25.20	98355	99.58
[10, 20[	279	0.28	98634	99.87
[20, 30[	101	0.10	98735	99.97
[30, 40[	22	0.02	98757	99.99
[40, 50[	4	0.00	98761	100.00
[50, 100[	4	0.00	98765	100.00
[100, 150[	0	0.00	98765	100.00
≥ 150	0	0.00	98765	100.00

© 2020 Izano MA et al. *JAMA Network Open.*

eTable 2. Distribution of Stabilized Weights Among Regimen 2 Followers, IPW (With Grace Period)

cum.IPAW	Frequency	%	Cumulative Frequency	Cumulative %
<0	0	0.00	0	0.00
[0, 0.5[	5962	3.43	5962	3.43
[0.5, 1[	70014	40.31	75976	43.74
[1, 10[	97563	56.17	173539	99.91
[10, 20[	129	0.07	173668	99.99
[20, 30[	15	0.01	173683	100.00
[30, 40[	3	0.00	173686	100.00
[40, 50[	1	0.00	173687	100.00
[50, 100[	1	0.00	173688	100.00
[100, 150[	0	0.00	173688	100.00
≥ 150	0	0.00	173688	100.00

© 2020 Izano MA et al. *JAMA Network Open.*

eTable 3. Distribution of Stabilized Weights Among Regimen 3 Followers, IPW (With Grace Period)

cum.IPAW	Frequency	%	Cumulative Frequency	Cumulative %
<0	0	0.00	0	0.00
[0, 0.5[	126	0.06	126	0.06
[0.5, 1[	82091	38.75	82217	38.81
[1, 10[	129563	61.15	211780	99.96
[10, 20[	68	0.03	211848	99.99
[20, 30[	12	0.01	211860	100.00
[30, 40[	1	0.00	211861	100.00
[40, 50[	1	0.00	211862	100.00
[50, 100[	1	0.00	211863	100.00
[100, 150[	0	0.00	211863	100.00
≥ 150	0	0.00	211863	100.00

© 2020 Izano MA et al. *JAMA Network Open.*

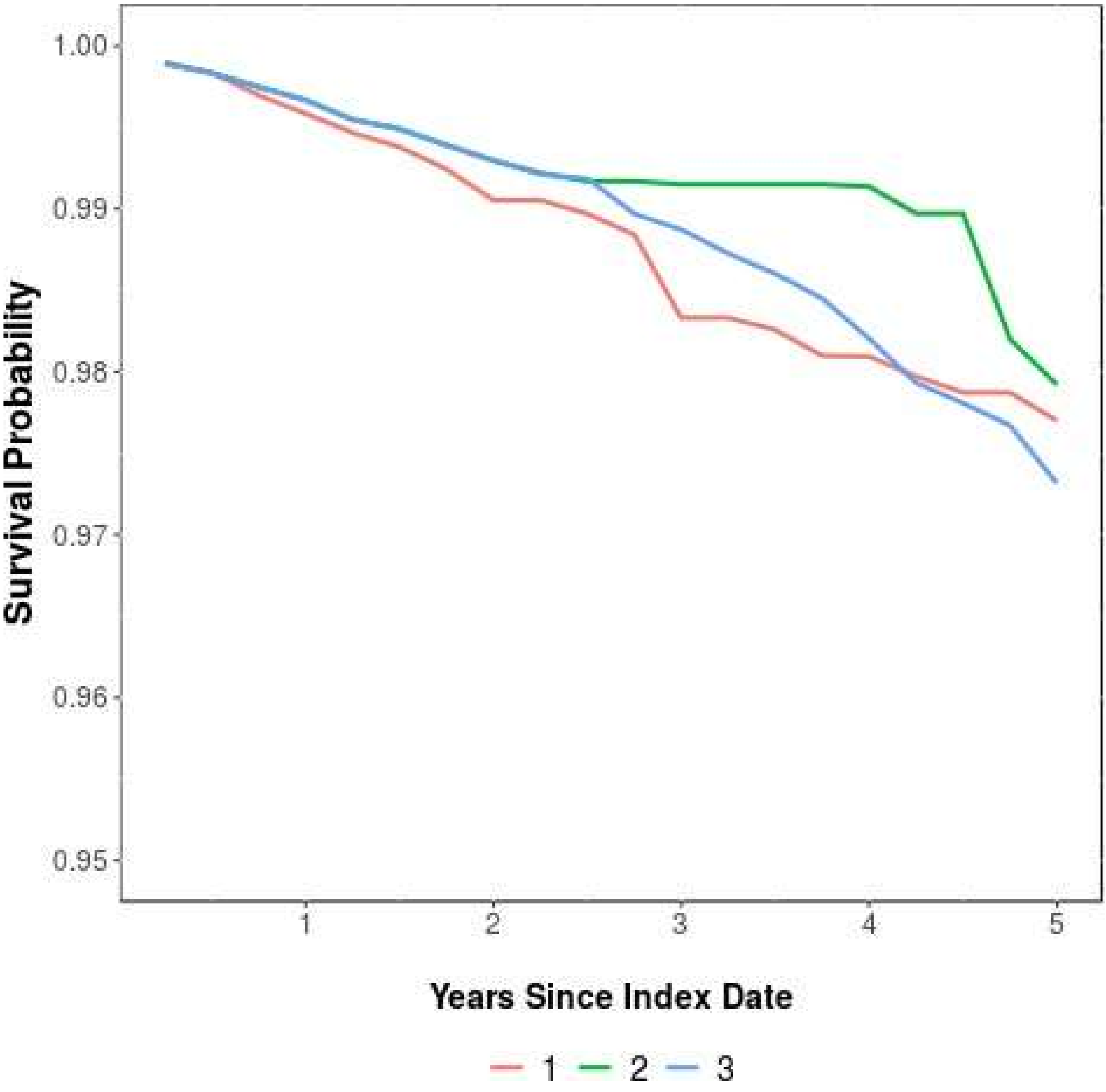

eFigure 3. IPW Survival Probabilities for the 3 Regimens of Interest (With Grace Period)
Regimen 1: discontinuation of BP at study entry; Regimen 2: discontinuation of BP at two years; Regimen 3: continuous BP exposure for 5 years

© 2020 Izano MA et al. *JAMA Network Open.*

eTable 4. IPW Estimated Risk Differences per 1000 Women Comparing Regimens of Interest (With Grace Period)

Year	3 vs. 1	2 vs. 1	3 vs. 2
1	-0.8 (-3.3, 1.6)	-0.8 (-3.3, 1.6)	0 (0, 0)
2	-2.4 (-7.7, 2.9)	-2.4 (-7.7, 2.9)	0 (0, 0)
3	-5.4 (-14.4, 3.6)	-8.2 (-17.1, 0.7)	2.8 (1.3, 4.3)
4	-1.1 (-10.9, 8.7)	-10.4 (-19.7, -1.1)	9.3 (6.3, 12.3)
5	3.8 (-7.4, 15.0)	-2.2 (-20.3, 15.9)	6.0 (-9.9, 22.0)

© 2020 Izano MA et al. *JAMA Network Open.*

eTable 5. Distribution of Unstabilized Weights Among Regimen 1 Followers, TMLE (With Grace Period)

cum.IPAW	Frequency	%	Cumulative Frequency	Cumulative %
<0	0	0.00	0	0.00
[0, 0.5[	0	0.00	0	0.00
[0.5, 1[	0	0.00	0	0.00
[1, 10[	86465	87.55	86465	87.55
[10, 20[	5513	5.58	91978	93.13
[20, 30[	2242	2.27	94220	95.40
[30, 40[	1265	1.28	95485	96.68
[40, 50[	813	0.82	96298	97.50
[50, 100[	1506	1.52	97804	99.03
[100, 150[	451	0.46	98255	99.48
≥ 150	510	0.52	98765	100.00

© 2020 Izano MA et al. *JAMA Network Open.*

eTable 6. Distribution of Unstabilized Weights Among Regimen 2 Followers, TMLE (With Grace Period)

cum.IPAW	Frequency	%	Cumulative Frequency	Cumulative %
<0	0	0.00	0	0.00
[0, 0.5[	0	0.00	0	0.00
[0.5, 1[	0	0.00	0	0.00
[1, 10[	167383	96.37	167383	96.37
[10, 20[	2359	1.36	169742	97.73
[20, 30[	703	0.40	170445	98.13
[30, 40[	436	0.25	170881	98.38
[40, 50[	467	0.27	171348	98.65
[50, 100[	1171	0.67	172519	99.33
[100, 150[	504	0.29	173023	99.62
≥ 150	665	0.38	173688	100.00

© 2020 Izano MA et al. *JAMA Network Open.*

eTable 7. Distribution of Unstabilized Weights Among Regimen 3 Followers, TMLE (With Grace Period)

cum.IPAW	Frequency	%	Cumulative Frequency	Cumulative %
<0	0	0.00	0	0.00
[0, 0.5[	0	0.00	0	0.00
[0.5, 1[	0	0.00	0	0.00
[1, 10[	197720	93.32	197720	93.32
[10, 20[	10497	4.95	208217	98.28
[20, 30[	2337	1.10	210554	99.38
[30, 40[	713	0.34	211267	99.72
[40, 50[	281	0.13	211548	99.85
[50, 100[	268	0.13	211816	99.98
[100, 150[	33	0.02	211849	99.99
≥ 150	14	0.01	211863	100.00

© 2020 Izano MA et al. *JAMA Network Open.*

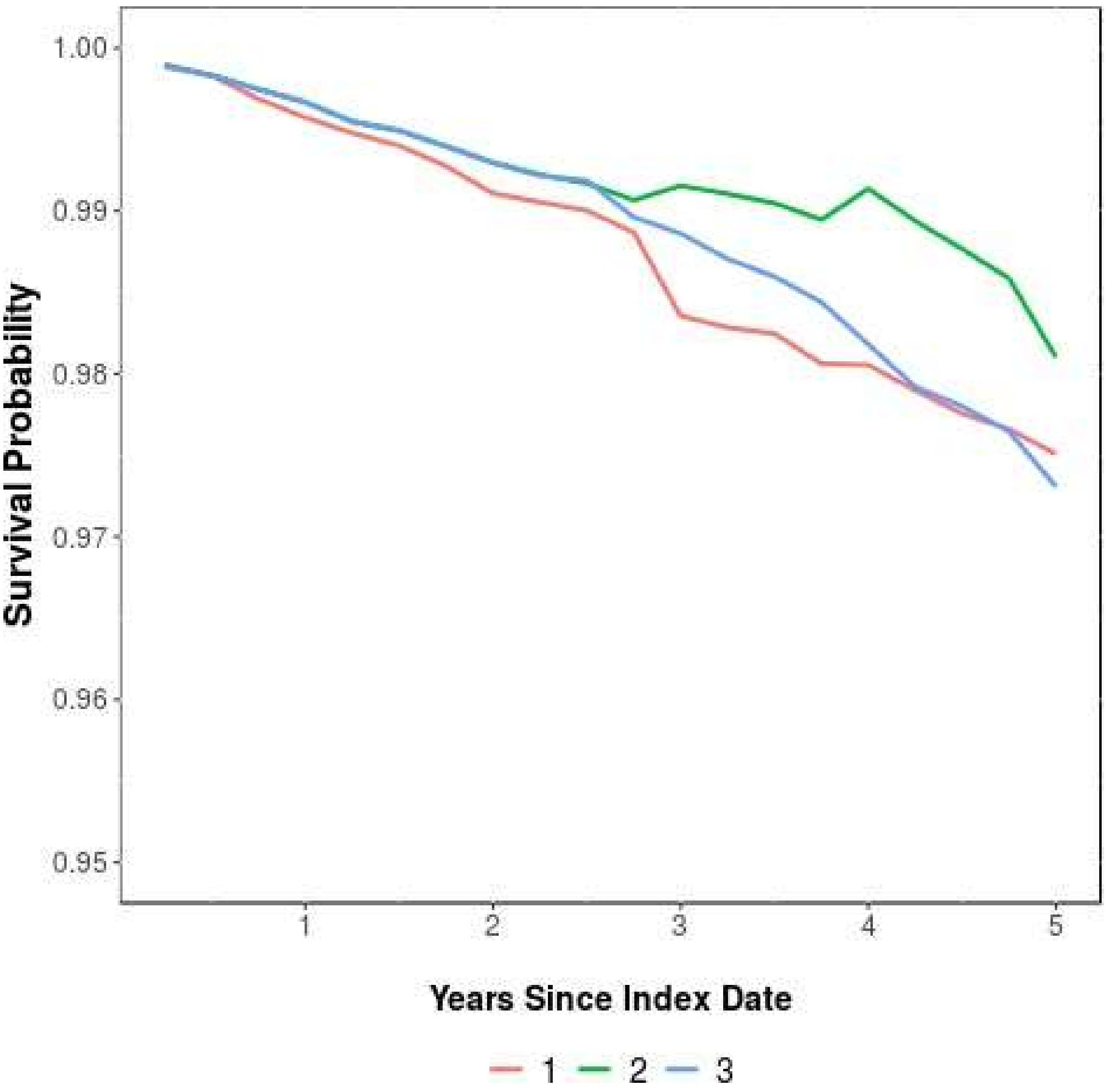

eFigure 4. TMLE Survival Probabilities for the 3 Regimens of Interest (With Grace Period)
Regimen 1: discontinuation of BP at study entry; Regimen 2: discontinuation of BP at two years; Regimen 3: continuous BP exposure for 5 years

© 2020 Izano MA et al. *JAMA Network Open.*

eTable 8. TMLE Risk Differences per 1000 Women Comparing Regimens of Interest (With Grace Period)

Year	3 vs. 1	2 vs. 1	3 vs. 2
1	-0.9 (-2.9, 1.0)	-0.9 (-2.9, 1.0)	0 (0, 0)
2	-1.9 (-7.0, 3.3)	-1.9 (-7.0, 3.3)	0 (0, 0)
3	-5.0 (-15.9, 5.8)	-8.0 (-18.6, 2.7)	2.9 (0.9, 5.0)
4	-1.3 (-13.5, 11.0)	-10.8 (-22.2, 0.5)	9.6 (4.9, 14.2)
5	2.0 (-12.9, 16.9)	-5.9 (-22.3, 10.5)	7.9 (-5.7, 21.5)

© 2020 Izano MA et al. *JAMA Network Open.*

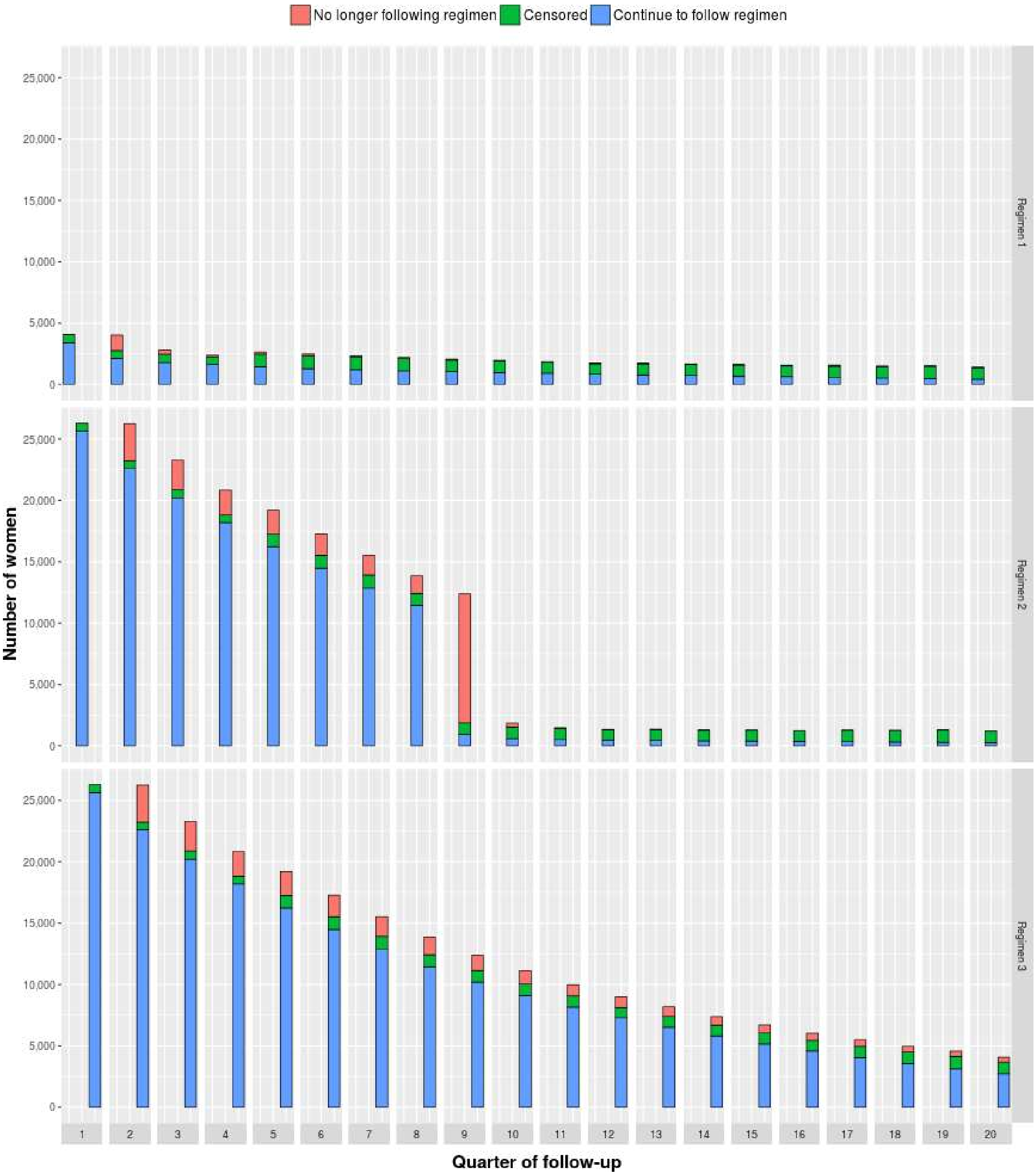

eFigure 5. Number of Women Who Continue to Follow, Are No Longer Following, or Were Censored from Regimens 1, 2, and 3 (Without Grace Period)

© 2020 Izano MA et al. *JAMA Network Open.*

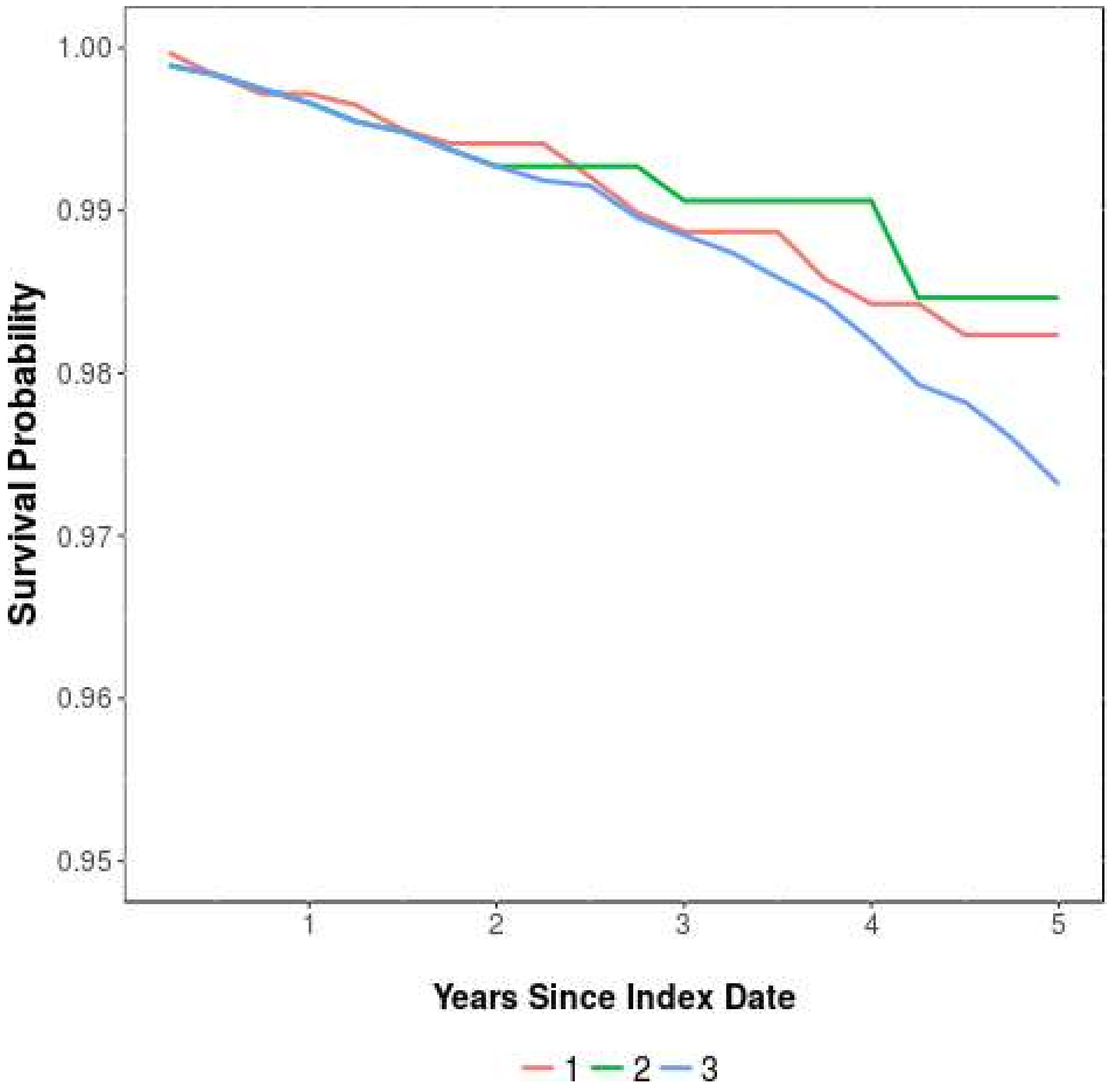

eFigure 6. Crude Survival Probabilities for the 3 Regimens of Interest (Without Grace Period)

Regimen 1: discontinuation of BP at study entry; Regimen 2: discontinuation of BP at two years; Regimen 3: continuous BP exposure for 5 years.

© 2020 Izano MA et al. *JAMA Network Open.*

eTable 9. Distribution of Stabilized Weights Among Regimen 1 Followers, IPW (Without Grace Period)

cum.IPAW	Frequency	%	Cumulative Frequency	Cumulative %
<0	0	0.00	0	0.00
[0, 0.5[	3733	16.60	3733	16.60
[0.5, 1[	7924	35.24	11657	51.84
[1, 10[	10534	46.84	22191	98.68
[10, 20[	208	0.92	22399	99.60
[20, 30[	48	0.21	22447	99.82
[30, 40[	25	0.11	22472	99.93
[40, 50[	4	0.02	22476	99.95
[50, 100[	11	0.05	22487	100.00
[100, 150[	1	0.00	22488	100.00
≥ 150	0	0.00	22488	100.00

© 2020 Izano MA et al. *JAMA Network Open.*

eTable 10. Distribution of Stabilized Weights Among Regimen 2 Followers, IPW (Without Grace Period)

cum.IPAW	Frequency	%	Cumulative Frequency	Cumulative %
<0	0	0.00	0	0.00
[0, 0.5[	788	0.54	788	0.54
[0.5, 1[	64362	43.83	65150	44.37
[1, 10[	81691	55.63	146841	100.00
[10, 20[	6	0.00	146847	100.00
[20, 30[	0	0.00	146847	100.00
[30, 40[	0	0.00	146847	100.00
[40, 50[	0	0.00	146847	100.00
[50, 100[	0	0.00	146847	100.00
[100, 150[	0	0.00	146847	100.00
≥ 150	0	0.00	146847	100.00

© 2020 Izano MA et al. *JAMA Network Open.*

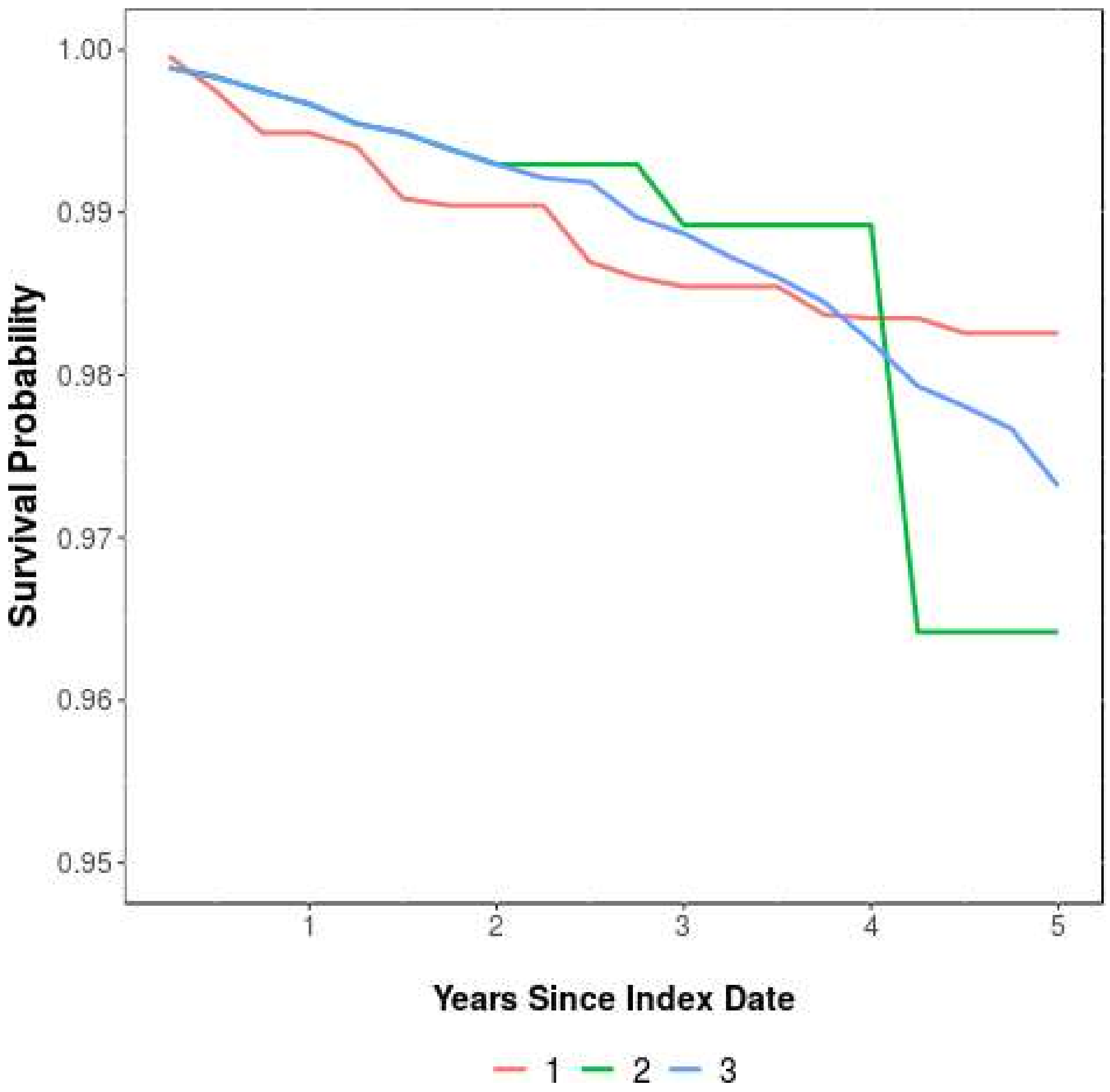

eFigure 7. IPW Survival Probabilities for the 3 Regimens of Interest (Without Grace Period)

Regimen 1 (discontinuation of BP treatment at study entry), Regimen 2 (discontinuation of BP treatment after two additional years of treatment), and Regimen 3 (remaining on treatment), respectively.

© 2020 Izano MA et al. *JAMA Network Open.*

eTable 11. IPW Estimated Risk Differences per 1000 Women Comparing Regimens of Interest (Without Grace Period)

Year	3 vs. 1	2 vs. 1	3 vs. 2
1	-1.8 (-7.0, 3.4)	-1.8 (-7.0, 3.4)	0 (0, 0)
2	-2.5 (-9.7, 4.6)	-2.5 (-9.7, 4.6)	0 (0, 0)
3	-3.3 (-12.2, 5.7)	-3.8 (-15.2, 7.7)	0.5 (-7.0, 8.0)
4	1.5 (-8.2, 11.1)	-5.7 (-17.5, 6.0)	7.2 (-0.7, 15.1)
5	9.4 (-1.3, 20.1)	18.4 (-27.7, 64.5)	-9.0 (-54.4, 36.4)

© 2020 Izano MA et al. *JAMA Network Open.*

eTable 12. Distribution of Unstabilized Weights Among Regimen 1 Followers, TMLE (Without Grace Period)

cum.IPAW	Frequency	%	Cumulative Frequency	Cumulative %
<0	0	0.00	0	0.00
[0, 0.5[	0	0.00	0	0.00
[0.5, 1[	0	0.00	0	0.00
[1, 10[	4827	21.46	4827	21.46
[10, 20[	6181	27.49	11008	48.95
[20, 30[	3345	14.87	14353	63.83
[30, 40[	2121	9.43	16474	73.26
[40, 50[	1317	5.86	17791	79.11
[50, 100[	2765	12.30	20556	91.41
[100, 150[	832	3.70	21388	95.11
≥ 150	1100	4.89	22488	100.00

© 2020 Izano MA et al. *JAMA Network Open.*

eTable 13. Distribution of Unstabilized Weights Among Regimen 2 Followers, TMLE (Without Grace Period)

cum.IPAW	Frequency	%	Cumulative Frequency	Cumulative %
<0	0	0.00	0	0.00
[0, 0.5[	0	0.00	0	0.00
[0.5, 1[	0	0.00	0	0.00
[1, 10[	141623	96.44	141623	96.44
[10, 20[	342	0.23	141965	96.68
[20, 30[	618	0.42	142583	97.10
[30, 40[	738	0.50	143321	97.60
[40, 50[	499	0.34	143820	97.94
[50, 100[	1490	1.01	145310	98.95
[100, 150[	705	0.48	146015	99.43
≥ 150	832	0.57	146847	100.00

© 2020 Izano MA et al. *JAMA Network Open.*

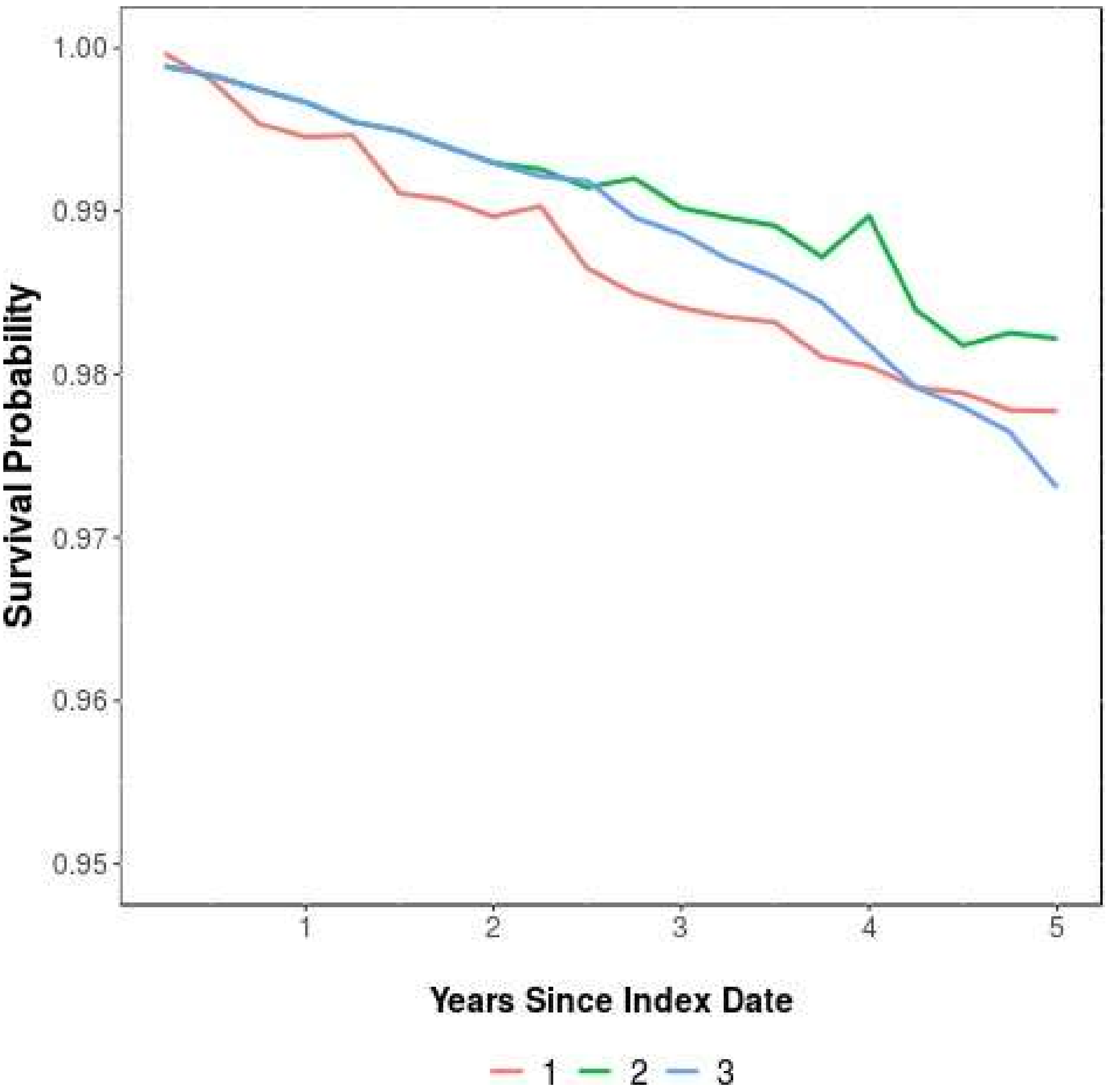

eFigure 8. TMLE Survival Probabilities for the 3 Regimens of Interest (Without Grace Period)

Regimen 1: discontinuation of BP at study entry continuous absence of BP exposure thereafter; Regimen 2: discontinuation of BP at two years and continuous absence of BP exposure thereafter; Regimen 3: continuous BP exposure for 5 years.

© 2020 Izano MA et al. *JAMA Network Open.*

eTable 14. TMLE Risk Differences per 1000 Women Comparing Regimens of Interest (Without Grace Period)

Year	3 vs. 1	2 vs. 1	3 vs. 2
1	-2.1 (-8.2, 3.9)	-2.1 (-8.2, 3.9)	0 (0, 0)
2	-3.3 (-13.1, 6.6)	-3.3 (-13.1, 6.6)	0 (0, 0)
3	-4.5 (-18.5, 9.4)	-6.1 (-21.4, 9.2)	1.6 (-5.4, 8.6)
4	-1.3 (-13.5, 11.0)	-9.2 (-25.5, 7.1)	7.9 (-0.2, 16.0)
5	4.7 (-12.6, 22.0)	-4.5 (-22.6, 13.7)	9.1 (-3.8, 22.1)

© 2020 Izano MA et al. *JAMA Network Open.*
